# Differential Expression of Candidate Virus Receptors in Human T Lymphocytes Prone or Resistant to Infection with Patient-Derived Hepatitis C Virus

**DOI:** 10.1371/journal.pone.0062159

**Published:** 2013-04-23

**Authors:** Mohammed A. Sarhan, Annie Y. Chen, Tomasz I. Michalak

**Affiliations:** Molecular Virology and Hepatology Research Group**,** Division of BioMedical Sciences, Faculty of Medicine, Health Sciences Center, Memorial University, St. John’s, Newfoundland and Labrador, Canada; Inserm, U1052, UMR 5286, France

## Abstract

Accumulated evidence implies that hepatitis C virus (HCV) infects not only the liver but also the immune system. A lymphocyte-specific CD5 molecule was recently identified as essential for infection of T cells with native, patient-derived HCV. To assess whether the proposed hepatocyte receptors may also contribute to HCV lymphotropism, expression of scavenger receptor-class B type 1 (SR-B1), claudin-1 (CLDN-1), claudin-6 (CLDN-6), occludin (OCLN), CD5 and CD81 was examined by real-time RT-PCR and the respective proteins quantified by immunoblotting in HCV-prone and resistant T cell lines, peripheral blood mononuclear cells (PBMC), primary T cells and their subsets, and compared to hepatoma Huh7.5 and HepG2 cells. SR-B1 protein was found in T and hepatoma cell lines but not in PBMC or primary T lymphocytes, CLDN-1 in HCV-resistant PM1 T cell line and hepatoma cells only, while CLDN-6 equally in the cells investigated. OCLN protein occurred in HCV-susceptible Molt4 and Jurkat T cells and its traces in primary T cells, but not in PBMC. CD5 was displayed by HCV-prone T cell lines, primary T cells and PBMC, but not by non-susceptible T and hepatoma cell lines, while CD81 in all cell types except HepG2. Knocking-down OCLN in virus-prone T cell line inhibited HCV infection, while *de novo* infection downregulated OCLN and CD81, and upregulated CD5 without modifying SR-B1 expression. Overall, while no association between SR-B1, CLDN-1 or CLDN-6 and the susceptibility to HCV was found, CD5 and CD81 expression coincided with virus lymphotropism and that of OCLN with permissiveness of T cell lines but unlikely primary T cells. This study narrowed the range of factors potentially utilized by HCV to infect T lymphocytes amongst those uncovered using laboratory HCV and Huh7.5 cells. Together with the demonstrated role for CD5 in HCV lymphotropism, the findings indicate that virus utilizes different molecules to enter hepatocytes and lymphocytes.

## Introduction

Hepatitis C virus (HCV) is a positive single stranded RNA virus that occurs as a symptomatic chronic infection in more than 170 million people. This infection represents a major health problem worldwide despite significant advancement in blood screening techniques [1], [2]. Currently, there are no vaccines preventing HCV infection, however new therapies show a significantly improved antiviral potency and augmented rates of HCV elimination, as measured by the detection of circulating HCV RNA by the presently available clinical assays [3]–[5]. Efforts to establish a robust HCV culture system have succeeded by transfecting human hepatoma Huh7 cells with a full-length HCV genome derived from a Japanese patient with fulminant hepatitis C (JFH-1), resulting in secretion of infectious HCV JFH-1 particles (HCVcc) [6]–[8]. This infection model and other HCV surrogate systems, such as HCV pseudoparticles (HCVpp) [9], [10], were applied to identify and/or to confirm molecules previously proposed to mediate HCV infection of hepatoma Huh7 cells and related cells lines which are expected to mimic normal human hepatocytes. As a result, tetraspanin CD81 [11], glycosaminoglycans [12], scavenger receptor class B type1 (SR-B1) [9], [13], and the tight junction (TJ) proteins such as claudin-1 (CLDN-1) [14], occludin (OCLN) [15], [16], and other molecules, such as epidermal growth factor receptor and ephrin receptor A2 [17] have been proposed as receptors determining HCV tropism to human hepatocytes. However, it remains uncertain to what degree these models and the molecules identified reflect events occurring in *in vivo* infection of hepatocytes with native virus.

Accumulated experimental and clinical evidence indicate that HCV infects not only hepatocytes but also cells in extrahepatic compartments, particularly those in the immune and the central nervous systems [18], [19]. In regard to infection of immune cells, HCV replication was shown in circulating T and B lymphocytes and monocytes from patients with symptomatic chronic as well as silently progressing persistent infections [20], [21]. The *ex vivo* susceptibility of primary T lymphocytes and certain T cell lines, such as Molt4 and Jurkat, to infection with native, patient-derived HCV and the ability of these cells to support the entire cycle of HCV replication in culture have also been shown [22]–[25]. The propensity of HCV to infect the host’s immune system is consistent with a significantly greater prevalence of lymphoproliferative disorders, such as mixed cryoglobulinemia and non-Hodgkin’s lymphoma, in patients infected with HCV [26]–[30]. In contrast to the several candidate receptors considered to be mediators of HCV hepatotropism, factors determining HCV lymphotropism are just being recognized. In this regard, a lymphocyte-specific CD5 glycoprotein, belonging to the scavenger receptor cysteine-rich family, has been recently identified to be essential for infection of human T lymphocytes with native, patient-derived HCV [25]. A contribution of CD81 to infection of T cells by the patient-derived virus has also been shown [23]–[25]. In the current study, the expression of SR-B1, CLDN-1, CLDN-6 and OCLN, in addition to CD5 and CD81, in HCV-prone and resistant T cell lines and in peripheral blood mononuclear cells (PBMC) and PBMC-derived primary T lymphocytes was investigated to assess whether they may contribute to infection of lymphocytes with authentic, patient-derived HCV and to compare their expression with that in JFH-1 strain-prone hepatoma Huh7.5 and JFH-1-resistant HepG2 cell lines. Based on the results of this comparative analysis, involvement of OCLN in facilitation of infection in T cell lines prone to naturally occurring virus was examined.

## Results

### Molt4 and Jurkat T Cells and Primary T Lymphocytes Demonstrate Comparable Susceptibility to Infection with Native HCV

**Figure 1:**
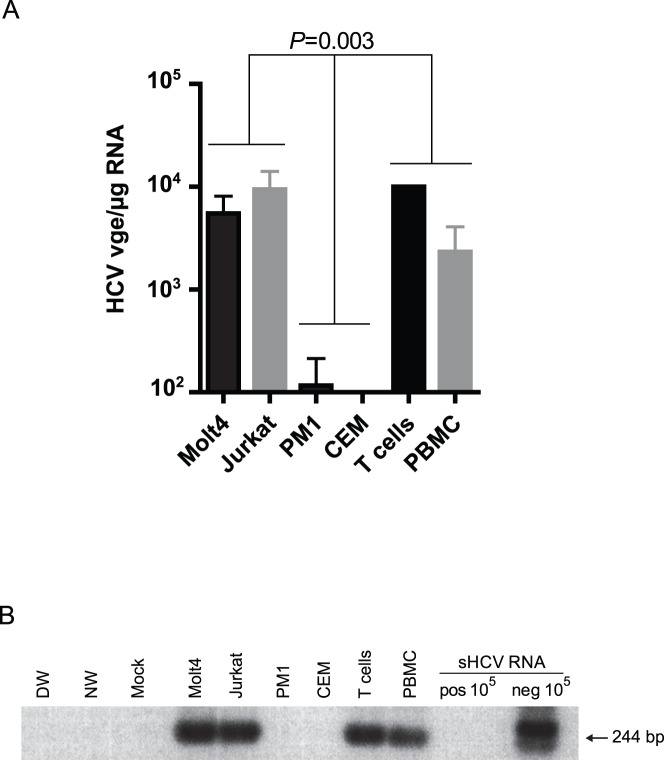
Identification of HCV in T cell lines, primary T lymphocytes and PBMC. Molt4, Jurkat, PM1 and CEM T cell lines, and primary T cells and total PBMC were exposed to plasma-derived, intact HCV and subsequently evaluated for the expression of HCV RNA positive strand by real-time RT-PCR and HCV RNA negative strand by RT-PCR/NAH. T cell lines were analyzed at day 7 post-infection. Primary T lymphocytes and PBMC isolated from healthy donors were exposed to the same virus as T cell lines and analyzed at day 14 post-infection, as described in Materials and Methods. (A) Quantification of HCV RNA positive strand. Data represent means from 3 separate experiments and are presented as HCV vge (copy) per µg total RNA. (B) Representative example of the detection of HCV RNA negative strand by RT-PCR/NAH in the cells from one of 3 experiments shown in A. Synthetic HCV RNA positive (pos) and negative (neg) strands at 10^5^ copies per reaction confirmed the assay specificity for detection of the virus RNA replication strand. Water amplified in direct (DW) and nested (NW) reactions and a mock extraction served as contamination controls. The positive signals showed the expected 244-bp oligonucleotide fragments.

Primary human T cells, PBMC from which the T cells were derived, and T cell lines, including HCV-prone Molt4 and Jurkat cells and HCV-resistant PM1 and CEM cells [25], were exposed to patient-derived HCV and subsequently examined for expression of HCV RNA positive and replicative strands. As the results showed (Fig. 1), Molt4 and Jurkat T cells displayed HCV RNA positive strand at levels comparable to those detected in primary T lymphocytes and PBMC (*P* = 0.6), while CEM cells where entirely nonreactive and PM1 cells only occasionally (*i.e.,* in one of 3 experiments) expressed the strand at a very low level (Fig. 1A). These results were compatible to those previously reported, including the observation that PM1 cells can become prone to HCV infection when activated [25].

Similarly as HCV RNA positive strand, the negative strand was detected at comparable levels in Molt4, Jurkat, primary T cells and PBMC following exposure to the same HCV inoculum. Figure 1B shows an example of the strand identification in the cells obtained in one of 3 experiments performed. In contrast, PM1 and CEM cells, which have been identified to be naturally resistant to infection with native HCV, were HCV RNA replicative strand nonreactive.

### Uniformly High Expression of CD81 and Aberrant Expression of SR-B1 Characterize T Cells Either Prone or Resistant to HCV Infection

**Figure 2:**
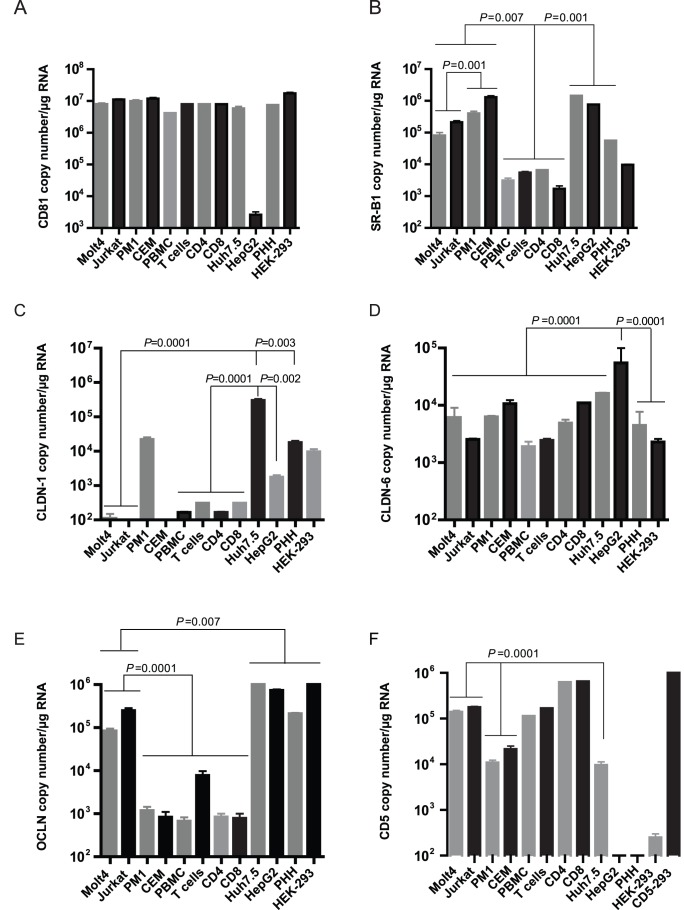
Quantification of expression levels of the genes encoding postulated HCV receptors in T cell lines, primary T lymphocytes, their CD4+ and CD8+ subsets, PBMC, hepatoma cell lines and human primary hepatocytes. Expression levels of: (A) CD81, (B) SR-B1, (C) CLDN-1, (D) CLDN-6, (E) OCLN, and (F) CD5. An equivalent of 50 ng DNAse-treated RNA served as template for real-time RT-PCR quantifications. The results represent means±SEM from 4 or more independent experiments and are presented as copies/µg total RNA.

Comparative analysis revealed that CD81 was ubiquitously expressed at similarly high levels in primary T cells, their CD4+ and CD8+ subsets and in all T cell lines investigated, independent of whether they were or were not susceptible to infection with HCV, as well as in Huh7.5, PHH, and control HEK-293 cells (Fig. 2A). The exception was HepG2 cells. Thus, CD81 mRNA levels typically ranged between 10^6^ and 10^7^ copies/µg total RNA, but HepG2 cells transcribed the gene at about 10^3^-fold lower levels (Fig. 2A).

Both HCV-susceptible and resistant T cell lines expressed SR-B1 at significantly higher (*P* = 0.007) levels than primary T cells, purified CD4+ or CD8+ T lymphocyte subsets or total PBMC. In general, primary T cells, their subsets and PBMC transcribed SR-B1 at the mean level of 3.9×10^3^± SEM 628 copies/µg total RNA, which was 2 to 3-fold lower than that detected in the T cell lines (Fig. 2B). Moreover, HCV-resistant PM1 and CEM cells transcribed significantly more (*P* = 0.001) SR-B1 compared to virus-prone Molt4 and Jurkat T cells. In regard to Huh7.5 and HepG2 hepatoma cells, no significant difference in SR-B1 expression was found (Fig. 2B). Further, PHH expressed about 10-fold less SR-B1 mRNA than Huh7.5 cells (*P* = 0.001) (Fig. 2B). There also was significantly lower transcription of SR-B1 in HCV-prone Molt4 and Jurkat cells (*P* = 0.0001) and in primary T cells, their subsets and PBMC (*P* = 0.0001) than in Huh7.5 cells.

It is of note that the expression levels of house-keeping ß-actin and HPRT genes were closely comparable in all cell types tested, except PHH. Thus, the ß-actin mRNA levels ranged between 10^7^ to 10^8^ copies/µg total RNA with no statistically significant variations. Approximately 10-fold lower transcription level was identified in PHH (data not shown). For HPRT, highly comparable mRNA levels were detected in all cell types examined, including PHH (data not shown).

### Expression of CLDN-1and CLDN-6 does not Coincide with T Cells Susceptibility to HCV Infection

CLDN-1 mRNA level was found to be high in Huh7.5 cells (3×10^5^± SEM 2×10^4^ copies/µg total RNA), while HepG2 and PHH showed 10 to 100-fold lower copy numbers (*P* = 0.002 and *P* = 0.003, respectively) (Fig. 2C). In contrast, HCV-susceptible Molt4 and Jurkat T cells essentially did not express CLDN-1, while PM1, but not CEM, transcribed CLDN-1 at a level greater than 10^4^ copies/µg total RNA. Primary T cells, their CD4+ and CD8+ subtypes and PBMC demonstrated CLDN-1 mRNA levels between 1×10^2^ and 1.5×10^2^ copies/µg RNA, which were significantly lower (*P* = 0.0001) than that detected in Huh7.5 cells (Fig. 2C).

In regard to CLDN-6 expression, no significant (*P* = 0.14) difference was found between HCV-prone and resistant T cell lines and Huh7.5 cells, while primary T cells, PBMC and PHH tended to transcribe approximately 10-fold lower levels than Huh7.5 cells (*P* = 0.01) (Fig. 2D). HepG2 cells expressed the highest (*P* = 0.0001) levels of CLDN-6 among the cells tested.

### High Expression of CD5 in Primary and Cultured T Cells and OCLN in T Cell Lines Coincides with Permissiveness to HCV

OCLN mRNA was expressed at high levels (10^5^–10^6^ copies/µg total RNA) in Huh7.5, HepG2, PHH and HEK-293 cells, while it was transcribed at approximately 10-fold lower (*P* = 0.007) levels in HCV-susceptible Molt4 and Jurkat cells (Fig. 2E). However, HCV-resistant PM1 and CEM cells, as well as PBMC and CD4+ and CD8+ T cell subsets transcribed OCLN at levels 10^2^–10^3^-fold lower (*P* = 0.0001) than the susceptible T cell lines (Fig. 2E). The total primary T cells demonstrated approximately 10-fold greater expression than their subsets or total PBMC, and the reason for this is not yet clear.

In regard to CD5, which transcription is lymphocyte restricted and the presence of CD5 protein has been identified to be essential for infection of T cells with HCV [25], it was confirmed that the gene expression was significantly greater (*P* = 0.0001) in HCV-prone primary T lymphocytes, their CD4+ and CD8+ subsets, PBMC and in Molt4 and Jurkat T cell lines, as well as in HEK-293 cells transfected with human CD5 serving as a control, than in virus-resistant PM1 and CEM T cells (Fig. 2F). Furthermore, PHH and HepG2 cells did not transcribe this gene, while hepatoma Huh7.5 cells displayed CD5 mRNA at the mean level of 9.6×10^3^± SEM 1.6×10^3^ copies/µg total RNA (Fig. 2F).

### Display of CD5 and OCLN Proteins Coincides with T Cell Permissiveness to HCV Infection

**Figure 3:**
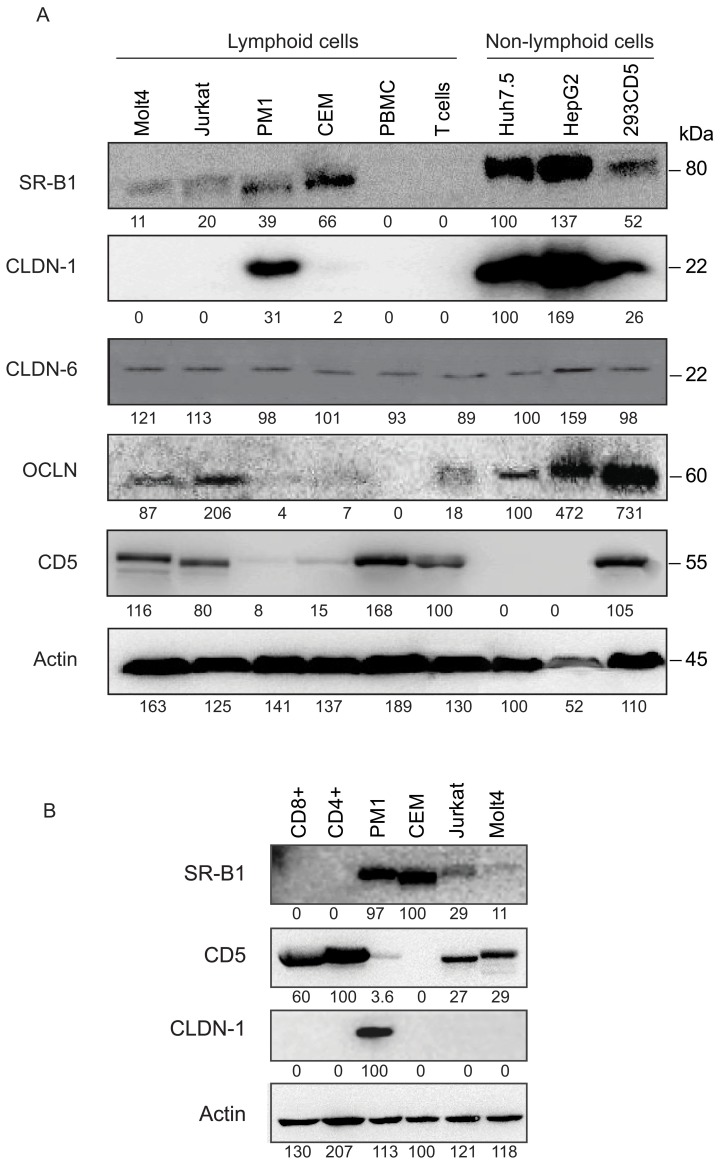
Differential display of HCV candidate receptor proteins in HCV-prone and resistant T cell lines, primary T lymphocytes and their subsets, PBMC, hepatoma cell lines and control HEK-293 cells transfected with human CD5. Proteins were separated at 25 µg per well by SDS-PAGE and after blotting probed with appropriate antibodies. Detection of β–actin served as a loading control. (A) Identification of SR-B1, CLDN-1, CLDN-6, OCLN, CD5 and β–actin in the cells investigated. The level of a given protein in a particular cell type is presented as a relative percentage of this protein signal detected in Huh7.5 cells that was taken as 100%, except CD5 protein for which display in primary T lymphocytes was taken as 100%. (B) Detection of SR-B1, CD5, CLDN-1 and β–actin in CD4+ and CD8+ subsets of primary T cell and in T cell lines investigated. The level of each protein in a particular cell type was presented as a relative percentage of the highest signal given by a given protein in any of the cell types examined that was taken as 100%.

T cell lines, primary T cells, total PBMC, as well as Huh7.5 and HepG2 cells were examined for the presence of the HCV candidate receptor proteins by probing with appropriate antibodies. In regard to SR-B1, the protein was displayed in HCV-resistant CEM (∼65% of the signal detected in Huh7.5 cells) and PM1 (∼40%), and at much lower levels in HCV-prone Molt4 (11%) and Jurkat (20%) T cells (Fig. 3A). In contrast, HepG2 displayed approximately 35% more of the protein and control HEK-293 cells transfected with CD5 about twice less than Huh7.5 cells. These results overall well corresponded to SR-B1 mRNA levels detected in the same cell types (Fig. 2B). Importantly, primary T cells, their CD4+ and CD8+ subsets and total PBMC were found to be SR-B1 protein non-reactive (Figs. 3A and 3B). This indicated that the SR-B1 mRNA detected at levels ranging between 10^3^ and 10^4^ copies/µg RNA as determined by real-time RT-PCR (Fig. 2B) was not translated to the amounts of the protein detectable by Western blotting (Fig. 3) or flow cytometry (data not shown).

In regard to the TJ proteins, CLDN-1 protein was detected only in HCV-resistant PM1 T cells (∼30% of the signal displayed by Huh7.5 cells) and in HepG2 hepatoma cells (∼170% of the signal identified in Huh7.5), and at a lower level (∼25%) by control HEK-293 cells transfected with CD5 (Figs. 3A and 3B). Notably, primary T cells, their CD4+ and CD8+ subsets and total PBMC, as well as HCV-prone Molt4 and Jurkat cells were entirely CLDN-1 protein negative (Figs. 3A and 3B). This was essentially consistent with CLDN-1 mRNA data which showed levels below 10^3^ copies/µg total RNA in primary T cells and undetectable levels in HCV-prone Molt4 and Jurkat T cells (Fig. 2C). On the other hand, CLDN-6 proteins were detected in all cell types at the levels which were comparable to those detected in Huh7.5 cells (Fig. 3A). Again, CLDN-6 mRNA levels well corresponded to the protein signals identified in individual cell types (Fig. 2D).

In regard to OCLN protein**,** HCV-susceptible Molt4 and Jurkat cells displayed approximately 90% and 205% of the protein signal detected in Huh7.5 cells, respectively (Fig. 3A). However, primary T lymphocytes presented the protein at about 10-fold lower level than Huh7.5 cells, while total PBMC were nonreactive. Furthermore, PM1 and CEM T cells exhibited trace levels of OCLN comparing to Huh7.5, *i.e.,* approximately 5% of the signal detected in Huh7.5 cells (Fig. 3A). HepG2 and control CD5-transfected HEK-293 demonstrated the protein at levels more than 4-fold and 7-fold greater than Huh7.5 cells, respectively (Fig. 3A). The OCLN protein signals appeared to well correspond to the gene expression levels detected in the same cell types (Fig. 2E).

CD5 protein was identified in HCV-susceptible Molt4 and Jurkat T cell lines, primary total T cells and PBMC (Fig. 3A) and in T cell subsets (Fig. 3B), and at trace levels in PM1 cell line. Huh7.5 and HepG2 cells were CD5 protein nonreactive, while control CD5-transfected HEK-293 cells showed the protein at a level comparable to that detected in primary T cells and T cell lines prone to HCV (Fig. 3A). There overall was a good agreement between CD5 protein display and the CD5 gene expression levels.

### HCV Infection Downregulates CD81 and OCLN but Augments CD5 and does not Change SR-B1 Expression in T Cells

**Figure 4:**
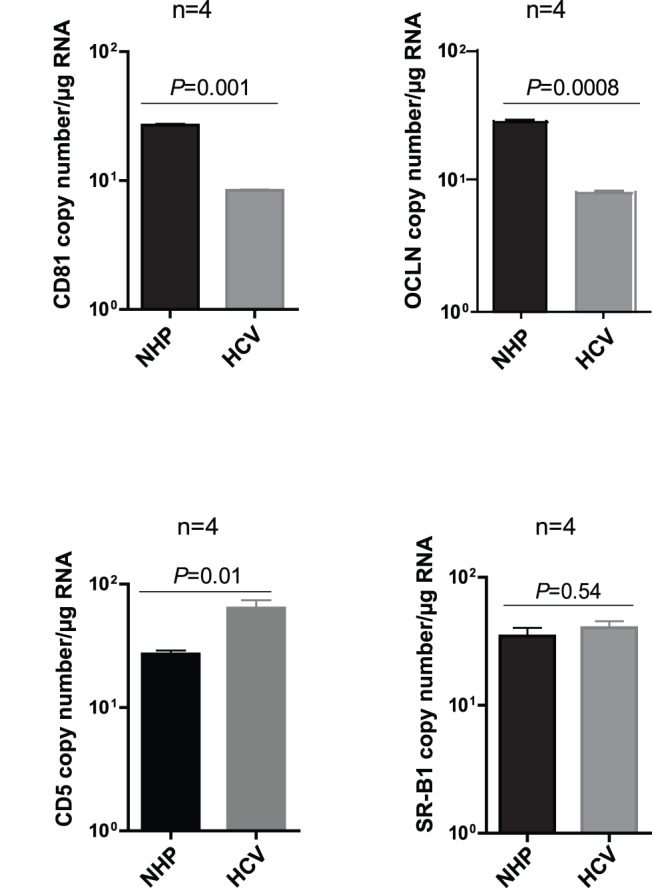
Expression levels of CD81, OCLN, CD5 and SR-B1 in Jurkat T cells infected with native, patient-derived HCV. Jurkat T cells infected with plasma-derived HCV or exposed to the equivalent amount of normal human plasma (NHP) were cultured for 5 days and then evaluated for the expression of (A) CD81, (B) OCLN, (C) CD5 and (D) SR-B1 by appropriate specific real-time RT-PCR assays. The results are presented as ratios of mean copy numbers/µg total RNA from 4 independent experiments of a given gene to actin.

Jurkat and Molt4 T cells exposed to native, patient-derived HCV or normal human plasma (NHP) as control were cultured for 5 days and subsequently examined for expression of CD81, OCLN, CD5 and SR-B1 by the appropriate real-time RT-PCR assays. The result showed a significant decrease in the level of transcription of CD81 (*P* = 0.001) and OCLN (*P* = 0.0008) in HCV-infected cells comparing to those exposed to NHP, as was illustrated for Jurkat cells in Figure 4. In contrast, CD5 mRNA level was significantly (*P* = 0.01) augmented in infected cells, while that of SR-B1 remained unchanged following infection (*P* = 0.54) (Fig. 4). This differential effect of HCV infection on the expression of CD81, OCLN and CD5, but not SR-B1 gene, provided supporting evidence that the respective proteins might be engaged in the infection process induced by HCV in T cells.

### Knockdown of OCLN Expression Inhibits HCV E2 Expression in HCV-prone T Cell Line

**Figure 5:**
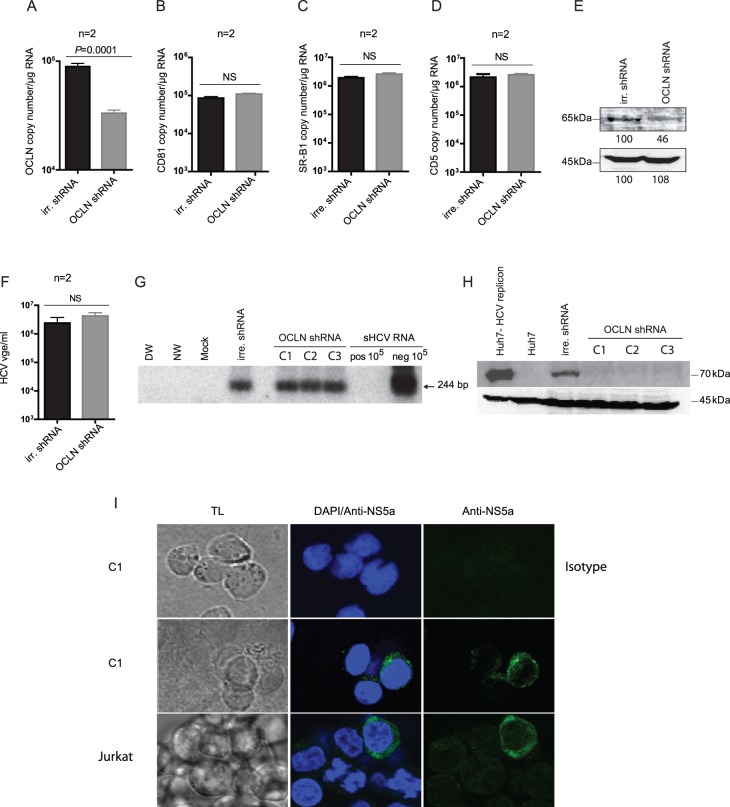
Effect of OCLN knockout on infection of Jurkat T cells with native, patient-derived HCV. Jurkat cells transduced with either OCLN-specific shRNA or control irrelevant (irr) shRNA were infected with plasma-derived HCV, as described in Materials and Methods. The levels of transcription of (A) OCLN, (B) CD81, (C) SR-B1 and (D) CD5 were evaluated at day 7 post-infection using appropriate real-time RT-PCR assays. The data are presented as mean copy numbers±SEM/µg total RNA from 2 independent experiments each evaluated in triplicate. (E) Western blot detection of OCLN protein in Jurkat cells transduced with either irr-shRNA or OCLN shRNA. Detection of β–actin served as a loading control. The level of OCLN protein in the cells transduced with OCLN shRNA is presented as a relative percentage of the OCLN protein signal detected in control cells that was taken as 100%. (F) Quantification of HCV RNA positive strand in Jurkat T cells transduced with either control irr-shRNA or three separate clones of OCLN shRNA and subsequently infected with wild-type HCV, as described in Materials and Methods. The data represent the mean copy (vge) numbers±SEM from 2 independent experiments each evaluated in triplicate. (G) Detection of HCV RNA replicative strand in Jurkat T cells transduced with either irr-shRNA or three different clones of OCLN shRNA (C1–C3) and infected with HCV, as indicated in (F). Synthetic HCV RNA positive (pos) and negative (neg) strands at 10^5^ copies/reaction served as the assay specific controls. DW, NW and a mock as described in the legend to Figure 1. (H) Detection of HCV E2 protein by Western blotting in Jurkat T cells transduced with irr-shRNA or with three OCLN shRNA clones (C1–C3) and then infected with HCV. Huh7 cells expressing HCV AB12-A2FL replicon and naïve Huh7 cells served as HCV E2 positive and negative controls, respectively. Detection of β-actin served as a loading control. (I) Detection of HCV NS5a protein by confocal microscopy in HCV infected Jurkat transduced with C1encoding OCLN-specific shRNA (C1) and in intact (untransduced) Jurkat cells infected with the same virus as a positive staining control. HCV-infected Jurkat transduced with C1 clone exposed to isotype antibody control instead of anti-NS5a served as a negative control. The cells were counterstained with DAPI to identify nuclei and examined under transmitted light (TL). The images were captured at X60 magnification.

Since the obtained data implied that OCLN may play a role in the facilitation of HCV infection in Molt4 and Jurkat T cell lines, although unlikely infection of primary T lymphocytes due to the presence of this protein at a trace level in the naturally circulating cells, the effect of the suppression of OCLN transcription on the infectivity of intact HCV towards Jurkat cells was investigated. In this regard, Jurkat cells were transduced with lentiviral particles encoding different human OCLN-specific shRNA, designated as C-1, C-2 or C-3, or with the control irrelevant shRNA, and then exposed to infectious HCV. The results showed that the level of OCLN expression was significantly (*P* = 0.0001) downregulated in OCLN-shRNA-transduced cells compared to control cells treated with irrelevant shRNA (Fig. 5A). It is of note that mRNA levels of CD81 (Fig. 5B), SR-B1 (Fig. 5C) and CD5 (Fig. 5D) were not affected by treatment with either OCLN-shRNA or irrelevant shRNA. Further, the display of OCLN protein was inhibited by ∼45% after transduction with OCLN-shRNA compared to the control cells (Fig. 5E). Most interestingly, although the knocking down of OCLN had no effect on the expression level of HCV RNA positive and negative (replicative) strands detected after exposure to virus, comparing to the control cells transduced with irrelevant shRNA (Figs. 5F and 5G), the display of HCV E2 protein was totally inhibited in the cells treated with OCLN-shRNA, but not in those transduced with irrelevant shRNA (Fig. 5H). However, display of HCV NS5a protein was not affected in cells with knockdown OCLN (Fig. 5I). This suggested that suppression of OCLN in fact severely affected HCV synthesis but on the posttranscriptional level likely via selectively affecting expression of E2 protein.

## Discussion

Despite that HCV is a hepatotropic virus and infection of hepatocytes is chiefly responsible for manifestations of hepatitis C, substantial experimental and clinical evidence indicate that HCV replication can also be supported by cells of the immune system, including T lymphocytes. The HCV RNA negative strand, representing virus genome replicative intermediate, viral proteins and virus sequences distinct from those occurring in the liver have been identified in PBMC, T and B lymphocytes, CD4+ and CD8+ T cell subsets, and in monocytes from patients with chronic hepatitis C (CHC) [20], [21], [30]–[35] and in persistent low-level infection continuing after clinically apparent sustained virological response to interferon (IFN) or IFN-ribavirin (IFN-RBV) therapy for CHC [21], [24], [34], [36]–[39]. HCV infection resulting in production of infectious virus has also been documented in *de novo* infected normal human T lymphocytes [22]–[24], cultured B cells [40], [41], and monocytes [20], [42]. A recent study from this laboratory has shown that naturally occurring HCV utilizes CD5, a lymphocyte unique glycoprotein, to infect T cells [25]. As in other chronic viral infections with noncytopathic viruses [43], [44], infection by HCV of immune privileged T cells may constitute an important mechanisms by which virus persists independently from its progeny multiplying in hepatocytes. In this regard, distinct HCV variants have been identified in the different compartments of virus occurrence, *i.e.,* liver and lymphomononuclear cells [20], [33], [36], [45], [46]. Further, HCV has been occasionally detected in circulating lymphoid cells but not in the liver, particularly in individuals with persistent low-level (occult) infection [33], [34], [36]. It has also been postulated that HCV variants inhabiting lymphoid cells can re-infect newly transplanted livers [32], [47], [48]. Also, HCV immune evasion and drug resistance might be, at least in part, a consequence of the virus ability to protractedly replicate in the lymphoid cell compartment, as in the case of hepatitis B virus infection in patients on suppressive antiviral therapy [49].

The host’s factors facilitating HCV infection of human cells were mainly investigated using HCV JFH-1 clone or HCVpp and human hepatoma cell lines. The JFH-1-Huh7.5 model provides a robust system in which JFH-1 HCVcc infect hepatoma Huh7.5 cells. However, these cells have a deficient innate immune response due to the lack of RIG-I expression, perturbed signaling and endocytic pathways, and dysfunctional mitochondria [50]–[55]. In addition, the genetic variance between JFH-1 clone and the naturally occurring HCV may further affect the properties and the nature of host factors mediating virus-cell interactions [56]. In this context, JFH-1 fails to infect human PBMC and primary T cells [57], which are known to be prone to infection with native HCV in both *in vivo* and *in vitro* conditions [21]–[23]. As well, JFH-1 clones do not infect T cell lines which are prone to patient-derived virus [58]. On the other hand, the authentic, patient-derived virus does not infect Huh7.5 cells [58].

In the current study, we found that Molt4 and Jurkat cells support HCV replication at approximately the same levels as primary T lymphocytes after exposure to the same HCV inocula. To recognize contribution of cellular factors to T cell susceptibility to HCV other than CD5 and CD81, we analyzed expression of selected HCV candidate receptors in HCV-prone and resistant T cells, and compared their expression to that in primary T cells and in JFH-1 strain-susceptible Huh7.5 and resistant HepG2 hepatoma cells. This analysis revealed that some of the postulated hepatocyte receptors are unlikely involved in determining HCV lymphotropism, while others may contribute to infectivity of cancer-derived T cell lines but unlikely normal primary T cells.

Considering distribution of the proposed HCV receptors in the lymphoid cells tested, the expression of tetraspanin CD81, which is known to require other cellular factors to mediate HCV cell entry [9], [59], was detected at comparable levels in all lymphocytic and non-lymphocytic cells examined, except HepG2 (see Fig. 2A). Although CD81 likely contributes to infection of T cells, based on the fact that antibodies against CD81 inhibited infection of primary T cells as well as Molt4 and Jurkat cells with native virus [23], [25], CD81 molecule is not the limiting factor of the infection since HCV-resistant PM1 and CEM T cells express CD81 at similar levels to cells prone to this virus (see Fig. 2A).

Another candidate receptor, SR-B1, is expressed at high levels in liver tissue and is considered an important HCV entry factor, as studies using soluble HCV E2 protein and HepG2 cells lacking CD81, but expressing SR-B1, showed. This interaction was found to be highly selective since neither mouse SR-BI nor the closely related human scavenger receptor CD36 was able to bind E2 [60]. However, although antibodies to SR-B1 markedly inhibited the binding of E2 protein or HCV-like particles to primary tupaia hepatocytes, they were not able to block infection with native, plasma-derived HCV [61]. Considering lymphocytes, a significantly (*P* = 0.007) lower expression of SR-B1 was detected in primary T cells, their CD4+ and CD8+ subsets and total PBMC than in T cell lines and Huh7.5, HepG2 and PHH. Moreover, HCV-resistant PM1 and CEM cells expressed significantly (*P* = 0.001) more SR-B1 than HCV-susceptible Molt4 and Jurkat T cells. In consequence, despite being susceptible to HCV, primary T cells, their subsets and PBMC did not display SR-B1 protein; therefore, the contribution of this protein to HCV lymphotropism can be with a high certainty excluded. This finding appears to be comparable to that reported by others where the interaction between soluble truncated HCV E2 protein and PBMC, B cells, monocytes or dendritic cells as targets was tested [62]. Taken together, the data indicate that while CD81 is likely involved in recognition of the host’s T cells by HCV, SR-B1 is not contributing to this process.

Although a pivotal contribution of CD5 to T cell tropism of HCV has been previously delineated [25], we re-analyzed expression of CD5 in the context of other HCV candidate receptors in the new set of experiments performed for the purpose of the current study. In general, the obtained data remained in agreement with those reported and confirmed that CD5 gene expression occurred at significantly (*P* = 0.0001) greater levels in HCV-susceptible than resistant T cells. This difference coincided with the detection of CD5 protein in HCV-prone but not in HCV-resistant T cells. In contrast to T cells, Huh7.5 and HepG2 cells did not display CD5 protein, as reported [25].

A role for TJ proteins in determining permissiveness of lymphoid cells to HCV remained uncertain. We compared the levels of CLDN-1, CLDN-6 and OCLN mRNAs and respective proteins in the cell types examined. CLDN-1, a protein previously reported to be an HCV entry factor mediating infection of hepatic cells, as it has been determined by examining interactions between HCVpp and HCVcc preparations and Huh7.5 cells [14], was expressed only by hepatoma cells and by lymphoma-derived PM1 T cells, which are resistant to HCV infection unless activated [25]. Unlike reported [14], HEK-293 cells were found to be CLDN-1 reactive, which might be due to a different cell clone or our more sensitive method of this protein detection. Thus, our results clearly showed that CLDN-1 is not involved in determining permissiveness of primary T lymphocytes to HCV. Further, CLDN-6 mRNA and its protein were identified at comparable levels in both HCV-susceptible and resistant cells, making their contribution to HCV infectivity of T lymphocytes also unlikely. In this regard, others have shown that human PBMC transcribe CLDN-6 but not CLDN-1 and that human hepatoma Bel7402 cell line was recognized by HCVpp despite that the cells did not express CLDN-1 [63]. This may suggest that CLDN-1 might be dispensable for infection of not only lymphocytes but also some non-lymphoid cells.

OCLN, which is another member of the TJ protein family, was shown to mediate infection of Huh7.5 cells by HCVcc and to be likely involved at the later stages of HCV entry [15]. Although OCLN expression was found to be significantly higher in hepatic cells, its mRNA and protein were detected in HCV-prone Molt4 and Jurkat T cells at much greater levels than in primary T cells and PBMC. To confirm potential relevance of OCLN to the facilitation or maintenance of HCV infection in virus-susceptible T cell lines, the effect of OCLN-knockout on the level of HCV replication in Jurkat T cells was investigated. While both HCV RNA positive and negative (replicative) strands were detected at comparable levels in the cells transduced with OCLN-shRNA or irrelevant-shRNA, virus E2 protein was detected only in the control cells treated with irrelevant-shRNA. This observation appears to be compatible to the previous report which showed no effect of OCLN silencing on the HCV RNA level in Huh7 cells carrying genomic or subgenomic HCV replicons [15]. In contrast to E2 protein, the display of HCV NS5a protein was not affected by knocking down of OCLN. The reason why HCV E2 protein, but not virus NS5a protein, was absent in the cells with silenced OCLN is not clear. It might be due to the interaction between E2 and OCLN and to the hypothetical possibility that OCLN facilitates translocation of the E2 protein to the appropriate intracellular compartment and/or prevents its premature release from the infected cell. In this regard, HCV E2 and OCLN have been found to co-localize in hepatocyte endoplasmic reticulum and co-immunoprecipitate [64]. Overall, the obtained data suggest that OCLN can selectively affect on the posttranscriptional level the HCV E2 protein expression in an infected T cell line. However, since OCLN protein was displayed at very low levels in primary T cells and not at all in PBMC, it may play a negligible role, if any, in HCV infectivity towards T cells *in vivo.*

In agreement with the previous findings showing downregulated expression of OCLN and CLDN-1 in Huh7 cells after infection with JFH-1 HCVcc [65], the level of OCLN and CD81 expression significantly decreased in Jurkat T cells infected with native HCV. Meanwhile, the transcription of CD5 became augmented and that of SR-B1 unchanged. These findings can be interpreted as an indication that HCV downregulates the display of OCLN and CD81 to prevent superinfection, as it has been postulated before [65]. This also provides supplementary evidence for the importance of these molecules in HCV infection of lymphocytes, at least in the case of the virus-susceptible T cell lines. On the other hand, upregulation of CD5 expression may protect cells from apoptosis and allow for more prolonged and robust HCV replication in the cells infected [66]–[68]. Further investigations will be required to assess validity of these hypotheses.

The data obtained in this study contribute to the better understanding of the nature of HCV lymphotropism, which remains a vaguely recognized property of naturally propagating virus. Overall, they are consistent with the notion that HCV utilizes a distinct set of cellular molecules to gain entry into human lymphocytes in comparison to that expected to mediate HCV tropism towards human hepatocytes. Among the molecules analyzed, only expression of CD5 is uniquely restricted to lymphocytes, implying a central role of this glycoprotein in HCV lymphotropism. However, no association between expression of SR-B1 or CLDN-1 and the susceptibility of T cells to infection with native HCV was found, while contribution of CD81 as a potential co-receptor was confirmed. Considering a surprisingly large number of cellular molecules implicated in HCV entry to hepatocytes, it cannot be excluded that yet other, not yet identified factors, are involved in the HCV tropism towards cells constituting the immune system.

## Materials and Methods

### Cell Lines, Primary Cells and Culture Conditions

Molt4 (CRL-1582) and Jurkat (TIB-152) T cell lines were provided by the American Type Culture Collection (ATCC, Manassas, Virginia, USA). PM1 cells were acquired from the National Institutes of Health AIDS Research and Reference Program (Rockville, Maryland, USA) and CCRF-CEM cells (CEM, ACC-240) from the Deutsche Sammlung von Mikroorganismen und Zellkulturen GmbH (Braunschweig, Germany). Molt4, Jurkat and CEM cell lines were originally derived from patients with acute T lymphoblastic leukemia [69], while PM1 from a patient with acute cutaneous T cell lymphoma [70]. The cells were cultured at 1×10^5^ cells/well in 5 ml of medium containing RPMI 1640 supplemented with 10% heat-inactivated fetal calf serum (FCS), 2 mM glutamine and 0.1 mM nonessential amino acids (Invitrogen Life Technologies, Burlington, Ontario, Canada). In some experiments, the cells were stimulated for 72 h with phorbol myristate acetate (PMA; Sigma-Aldrich, Oakville, Ontario, Canada) at 50 ng/ml in the presence of 500 ng/ml ionomycin (Sigma-Aldich), as reported [25].

Total PBMC were isolated from two healthy donors by gradient centrifugation in Ficoll-HyPaque (Pharmacia Biotech Inc., Baie-D’Urfé, Quebec, Canada) [37]. Primary T lymphocytes were affinity-purified from monocyte-depleted PBMC by negative selection using MACS magnetic microbeads (Miltenyi Biotec, Auburn, California, USA), as reported [25], [39]. CD4+ and CD8+ T cell subsets were isolated from PBMC by affinity chromatography using AutoMacs Pro-Separator system (Miltenyi Biotec) following the manufacturer’s instruction. T cells and their subsets were 97–98% pure by flow cytometry analysis. For some experiments, PBMC and affinity-purified primary T cells were stimulated with 5 µg/ml phytophemagglutinin (PHA; Sigma-Aldrich) in the presence of 20 IU/ml human recombinant interleukin-2 (IL-2; Roche Molecular Diagnostics, Pleasanton, California, USA), as described [21], [37].

HCV replicon AB12-A2FL Huh7 cell line, containing full-length HCV genotype 1b, was provided by Drs Christopher Richardson and Joyce Wilson formerly from Ontario Cancer Institute, University of Toronto, Canada. The human hepatoma Huh7.5 cells were provided by Dr. Takaji Wakita from National Institute of Infectious Diseases, Shinjuku, Japan and Dr. Rodney Russell from Memorial University of Newfoundland, St. John’s, NL Canada. Human hepatoma HepG2 (HB-8065) and human embryonic kidney 293 fibroblasts (HEK-293; CRL-1573) cell lines were supplied by ATCC. The cells were maintained in DMEM medium supplemented with 10% FCS. PHH isolated by microperfusion of a healthy portion of the liver of a 58-year old male donor were purchased from Dominion Pharmakine (Derio-Bizkaia, Spain) [71].

### HCV Infection Assay

Cultured T cell lines, either intact or stimulated with PMA/ionomycin for 72 h at 1×10^5^ cells/well, supplemented with 2 ml of culture medium in 6-well plates were exposed for 24 h to ∼1×10^5^ virus genome copies (virus genome equivalents, vge) of HCV genotype 1a-positive plasma pool prepared from patients with CHC who were antiviral treatment naive. Infectivity of the pool was established in preliminary experiments using Molt4 and Jurkat T cell lines and PHA-stimulated primary T lymphocytes, as previously reported [23], [25]. Cells exposed under identical conditions to an equivalent volume of NHP served as negative controls. After exposure to HCV or NHP, the cells were extensively washed, suspended in fresh culture medium and cultured for 4–7 days post-infection (d.p.i.), as reported [25]. The study protocol was approved by the local Human Investigation Committee.

### Silencing of OCLN by RNA Interference

Lentiviral transduction particles encoding different human OCLN-specific shRNA (clones: TRCN0000158463, TRCN0000158804, TRCN0000159413, TRCN0000159613 and TRCN0000159771; designated as C-1 to C-5) with a titer between 2.9×10^7^ and 3.9×10^7^ infectious virus and 4.6×10^7^ non-target control transduction particles (#SHC001V; designated as irr) were prepared by Sigma-Aldrich. 1×10^3^ Jurkat cells in 100 µl RPMI 1640 medium was transduced at 4 multiplicity of infection (MOI) in 96-well plates in the presence of 2.5 µg/ml polybrene (Sigma-Aldrich). Cells were spun down at 4°C for 10 min at 2,000 rpm, incubated at 37°C for 24 h, and allowed to revive in fresh medium for another 24 h. Transduced Jurkat cells were selected in the presence of 2 µg/ml puromycin (Sigma-Aldrich) for 7 days. The level of OCLN expression was determined by real-time RT-PCR and Western blotting (see below). Only C-1, C-2 and C-3 were used in subsequent experiments, since C-4 and C-5 were found to be toxic to Jurkat T cells. Subsequently, Jurkat cells transduced with C-1, C-2 or C-3 or with the control scrambled shRNA, and the nontransduced cells were used as targets in HCV infection following the procedure described above.

### RNA and Transcription to cDNA

Total RNA was extracted using Trizol (Invitrogen Life Technologies, Burlington, Ontario, Canada). RNA used for quantification of cellular genes was treated with DNAse, as reported [38]. cDNA was transcribed with Moloney murine leukemia virus reverse transcriptase (RT) (Invitrogen), as previously described [25], [37].

### Detection of HCV

HCV RNA positive and negative (replicative) strands were identified using cDNA derived from 1 or 2 µg and 2 or 3 µg, respectively, of total RNA and the strand-specific amplification conditions reported in detail previously [25], [37]. The specificity of the signal detection and validity of controls were routinely confirmed by nucleic acid hybridization (NAH) with ^32^P-labeled recombinant HCV 5′-untranslated region-E2 fragment (rHCV UTR-E2) as a probe [37]. The sensitivity of RT-PCR/NAH assay for HCV RNA-positive strand identification was <10 vge/ml (<2 IU/ml) or <5 vge/µg of total RNA, while that for HCV RNA-negative strand was 25–50 vge/µg of total RNA, as reported [25], [37].

### Cloning of SR-B1 and TJ Gene Sequences

To facilitate quantification of the expression levels of SR-B1 and TJ genes in the cells examined, cDNA transcribed from human PBMC or liver was amplified by PCR with primers shown in Table 1. Amplified fragments were cloned into the dual promoter vector PCRII using the TOPO TA cloning system (Invitrogen) and then excised and sequenced in both directions to confirm the gene specificity. The cloned fragments were used as quantitative and the sensitivity standards in real-time RT-PCR assays. Human CD5 and CD81 gene fragments were generated in the previous study [25].

**Table 1:** Primer sequences used for real-time RT-PCR analysis.

Gene	Forward primer	Reverse primer
CD5	5′-TCAAGCGTCAAAAGTCTGCC-3′	5′-AGCCACACTGGAGGTTGTTG-3′
CD81	5′-ACAAGGACCAGATCGCCAAG-3′	5′-AGTCAAGCGTCTCGTGGAAG-3′
OCLN	5′-TGCATGTTCGACCAATGC-3′	5′-AAGCCACTTCCTCCATAAGG-3′
SR-B1	5′-TCGCAGGCATTGGACAAACT-3′	5′-CTCCTTATCCTTTGAGCCCTTTT-3′
CLDN-1	5-TACTCCTATGCCGGCGACA-3′	5′-GACATCCACAGCCCCTCGT-3′
CLDN-6	5′-AGAAGGATTCCAAGGCCCG-3′	5′-GATGTTGAGTAGCGGGCCAT-3′
β-Actin	5′-CATCCTCACCCTGAAGTACC-3′	5′-CATACTCCTGCTTGCTGATCC-3′
HPRT	5′-TGACACTGGCAAAACAATGCA-3′	5′-GGTCCTTTTCACCAGCAAGCT-3′

### Real-time RT-PCR

The expression of SR-B1, CLDN-1, CLDN-6, OCLN, CD81 and CD5 was quantified by specific real-time RT-PCR assays during 40 cycles using the LightCycler 480 (Roche Diagnostics, Mannheim, Germany). Reactions were performed in 10-µL volumes, each containing 2 µL cDNA derived from 50 ng DNAse-treated RNA using primer pairs shown in Table 1. Annealing temperature of 56°C were used for amplification of OCLN, CD81, β-actin and hypoxanthine phosphoribosyltransferase (HPRT), while 60°C annealing temperature for amplification of SR-B1, CLDN-1, CLDN-6 and CD5. Expression of genes of interest was evaluated in samples obtained from at least 4 separate experiments. β-actin and HPRT were used as loading controls. The sensitivity of the real-time RT-PCR assays was determined using 10-fold, serial dilutions of the cloned fragments of the respective genes and was found to be between 10 to 10^2^ copies/reaction. In regard to HCV RNA positive strand quantification, the copy numbers were enumerated by real-time RT-PCR using 10-fold serial dilution of rHCV UTR-E2, as described previously [21].

### Western Blotting

Cells were treated with ice-cold RIPA buffer (1% NP-40, 0.5% DOC, 0.1% SDS, 150 mmol/L NaCl in 50 mmol/L Tris, pH 8.0; Sigma-Aldrich) and cellular debris removed by centrifugation. Proteins were separated by sodium dodecyl sulfate-polyacrylamide gel electrophoresis (SDS-PAGE) at 25 µg protein per lane and then blotted onto a nitrocellulose membrane (Amersham Biosciences, Piscataway, New Jersey, USA) by wet transfer using the Bio-Rad SD cell system (Bio-Rad Laboratories, Mississauga, Ontario, Canada) [72]. The blots were incubated with 5% skimmed milk in Tris-buffered saline, pH 7.4, for 1 h at room temperature and exposed overnight at 4°C to working dilutions of appropriate antibodies diluted in the same blocking buffer. Mouse monoclonal antibodies (mAb) to SR-B1 (clone 25/CLA-1) and OCLN (clone 19) were purchased from B.D. Biosciences (Mississauga, Ontario, Canada), rabbit antibodies to CLDN-1 were from Invitrogen, while rabbit antibodies to CLDN-6 (ab75055) and CD5 (ab52964) were acquired from Abcam Inc. (Cambridge, Massachusetts, USA). In some instances, the presence of HCV envelope protein in infected lymphocytes and in control AB12-A2FL Huh7 cells was detected with anti-E2 mouse mAb provided by Dr. Arvind Patel, University of Glasgow, Glasgow, United Kingdom, as reported [25]. Detection of β-actin with specific rabbit mAb (Sigma-Aldrich) served as protein loading control. Reactions were developed with horseradish peroxidase-conjugated goat anti-rabbit or goat anti-mouse IgG F(ab’)_2_ antibodies (Jackson ImmunoResearch, West Grove, Pennsylvania, USA) and visualized using an ECL detection kit (Sigma-Aldrich). Protein signals were quantified by densitometry using the computer-assisted image processing application ImageJ from the National Institute of Health [73]. For comparative evaluations, the density of the protein signals detected in Huh7.5 cells were taken as 100%, except for CD5 protein for which the signal detected in primary T cells was used as 100% or unless otherwise indicated.

### Confocal Microscopy

To detect HCV NS5a protein, cells were fixed with 2% paraformaldehyde, permeabilized with 0.25% saponin, and then exposed to either mouse anti-HCV NS5a mAb (Chemicon International, Temecula, California, USA) or appropriate isotype mAb control (BD Biosciences) overnight at 4°C, washed and then exposed to Cy2-labeled donkey anti-mouse antibody (Jackson ImmunoResearch) on ice for 60 min. Subsequent washing and counterstaining with 4′,6-diamidino-2-phenylindole (DAPI; 0.1 µg/ml) (Sigma-Aldrich) were done and slides were examined under an Olympus BX50WI microscope with a FlouView FV300 confocal system (Olympus America Inc., Melville, New York State, USA) as previously reported [25].

### Statistical Analyses

Results were analyzed by a one way analysis of variance or unpaired Student *t* test with Welch’s correction using GraphPad Prism software (GraphPad Software, Inc., San Diego, California, USA). Differences between experimental conditions were considered to be significant when two-sided *P* values were below or equal to 0.05.
